# UV radiation limited the expansion of cyanobacteria in early marine photic environments

**DOI:** 10.1038/s41467-018-05520-x

**Published:** 2018-08-06

**Authors:** Aleksandra M. Mloszewska, Devon B. Cole, Noah J. Planavsky, Andreas Kappler, Denise S. Whitford, George W. Owttrim, Kurt. O Konhauser

**Affiliations:** 1 Earth Sciences Department, University of Toronto, Toronto, M5S 3B1 ON Canada; 2 Applied Geosciences, University of Tübingen, Tübingen, 72074 Germany; 3 Earth and Atmospheric Sciences, University of Alberta, Edmonton, T6G 2E3 AB Canada; 4 Department of Geology and Geophysics, Yale University, New Haven, 06511 CT USA; 5 Biological Sciences, University of Alberta, Edmonton, T6G 2E9 AB Canada

## Abstract

Prior to atmospheric oxygenation, ecosystems were exposed to higher UV radiation fluxes relative to modern surface environments. Iron–silica mineral coatings have been evoked as effective UV radiation shields in early terrestrial settings. Here we test whether similar protection applied to planktonic cyanobacteria within the Archean water column. Based on experiments done under Archean seawater conditions, we report that Fe(III)–Si-rich precipitates absorb up to 70% of incoming UV-C radiation, with a reduction of <20% in photosynthetically active radiation flux. However, we demonstrate that even short periods of UV-C irradiation in the presence of Fe(III)–Si precipitates resulted in high mortality rates, and suggest that these effects would have persisted throughout much of the photic zone. Our findings imply that despite the shielding properties of Fe(III)–Si-rich precipitates in the early water column, UV radiation would continue to limit cyanobacterial expansion and likely had a greater effect on Archean ecosystem structure before the formation of an ozone layer.

## Abstract (web summary)

The means by which planktonic cyanobacteria were able to persist through the Archean despite high fluxes of UV radiation are unclear. Here, the authors show that Fe(III)-Si rich precipitates in the Archean photic zone could have provided early planktonic cyanobacteria an effective shield against UV-C radiation.

## Introduction

Solar ultraviolet (UV) radiation is a key factor controlling the habitability of planetary surface environments and regulating the course of life’s evolution. Prior to 2.4 Ga and the development of a protective stratospheric ozone layer^1^, the flux of UV radiation—in particular UV-C wavelengths—reaching the surface of the Archean ocean was orders of magnitude higher than today^2,3^. As a result, rates of DNA damage to unprotected organisms could have limited the size and scope of the biosphere. In order to survive, photosynthesizing microorganisms, such as cyanobacteria, would have required either the evolution of DNA repair and protein turnover mechanisms to reverse the damage caused by UV radiation or an environmental mechanism to protect them against the harmful effects of radiation^4^. Additionally, renewed interest in the ability of life to tolerate high radiation environments has been sparked by the recent discovery of Earth-sized rocky planets within the habitable zones of a number of nearby M-dwarf stars (e.g., TRAPPIST-1^5^, Proxima Centauri^6^, among others). These stars have substantially different stellar spectra compared to the sun, and the planets reside much closer to their stars, likely resulting in elevated UV radiation fluxes on these exoplanetary surfaces^5,6^. The results presented here highlight the importance of understanding environmental conditions on the early Earth as a case study to better quantify the potential for the emergence of substantial biospheres on exoplanetary surfaces likely to experience elevated UV radiation levels.

Modern cyanobacteria have mechanisms to counteract damage by UV radiation such as DNA excision repair and photoreactivation^7^, DNA repair systems like the SOS response^8^, detoxifying enzymes and pigments^9^, and UV radiation-absorbing sunscreen molecules^10^. However, these systems can rapidly become overwhelmed by sustained, high doses of UV radiation^11^, and some strains of cyanobacteria are often too small to accommodate enough sunscreen molecules for effective protection^12^. For benthic microorganisms, which live an aggregate lifestyle, mineralized coatings and extracellular sheaths provide physical protection from UV radiation^13^. Many of these organisms are able to migrate downward into the sediment or microbial mat and use exposure to UV radiation as an environmental cue^14^. While some planktonic cyanobacteria have the ability to adjust buoyancy by means of gas vacuoles or carbon ballasting^15^, regions of strong upwelling (where productivity will be highest) or wind-induced mixing within the upper tens of meters of the water column may still overcome those adjustments and force phototrophic bacteria seeking refuge within deeper parts of the photic zone to intermittent periods of exposure to high levels of UV radiation^16^.

Absorption of UV radiation in the oceans today occurs as a result of water’s optical and hydrodynamic properties as well as by dissolved and particulate matter suspended in the water column^17^. Within modern coastal habitats, plant-derived humic substances, either colored dissolved organic matter (CDOM) or particulate organic matter, play dominant roles in the attenuation of UV radiation^18^. Due in large part to the presence of CDOM in the water column, the diffuse attenuation coefficient of UV radiation for natural waters not only tends to vary greatly, but also to differ significantly from the ideal value for water^19^. In the open ocean, UV radiation can penetrate deep into the mixed layer of the water column. For example, in clear ocean waters of the Mediterranean Sea, UV radiation can cause phytoplankton mortality down to 26 m^20^. The difficulty in accurately predicting the penetration depth of UV-C in early seawater based on attenuation coefficients of modern seawater lies in the fact that today UV-C exposure is largely attenuated prior to reaching the Earth’s surface^3^. However, the absence of plant-derived humic substances and a protective ozone layer in the Archean may have allowed high-intensity UV radiation (including UV-C wavelengths) to reach greater depths in the ancient water column^2^. Cockell^2^ used a radiative transfer model that incorporated diffuse attenuation coefficients for the clearest modern ocean waters to predict that UV-B and UV-C radiation would have occurred at sufficient intensity to cause significant UV radiation-induced DNA damage throughout the mixed layer of the Archean ocean. Under these conditions it is likely that UV radiation would have been an even greater obstacle for early cyanobacteria than for their modern descendants.

Although a late origin for cyanobacteria has been proposed^21^, multiple lines of evidence suggest that planktonic populations of cyanobacteria evolved in and were able to persist through the Archean despite high fluxes of UV radiation^22–24^. An ancestral lineage and deep origin of planktonic cyanobacteria is inferred from the conservation of the GvpA and GvpC proteins that make up the gas vesicle walls designed to prevent them from sinking out of the euphotic zone^15^. Moreover, most mat-forming cyanobacteria have a free-floating stage in their life cycle which occurs in intertidal and near-shore areas of the coastal ocean and enables the colonization of new habitats^25,26^. So, although there is evidence for planktonic cyanobacteria in early marine environments from 2.7 Ga or earlier^22–24^, the means by which they overcame Archean UV radiation fluxes, and the scope of the effect of these fluxes on the biosphere remains uncertain.

Various dissolved and particulate iron species have been shown to be efficient absorbers of UV radiation, beginning with the pioneering study of Olson and Pierson^27^. The Archean oceans are generally agreed upon to have been dominated by anoxic-rich and iron-rich (ferruginous) conditions. Archean-dissolved marine Fe(II) concentrations are estimated to have been on the order of tens-to-hundreds of micromolar^24,28^, consistent with modern system Archean analogs^29^. In surface waters, a significant portion of this Fe(II) was oxidized to ferric oxyhydroxides, such as ferrihydrite, (Fe^+3^)_2_O_3_•0.5H_2_O_,_ by anoxygenic photoferrotrophic bacteria^30,31^, which likely sedimented to the seafloor within a matter of days^32^. Iron isotope signatures in Archean banded iron formations (BIF) imply variable Fe(II) oxidation rates ranging from partial^33^ to near-complete^34^ within the ancient water column. Indeed, active communities of photoferrotrophic bacteria residing within modern, deep light-limited communities oxidize Fe(II) at a rate of up to 0.45 mol/m^2^/yr^32^, while Fe(II) oxidation rates of up to 50 mol/m^2^/yr have also been observed within the upper 15 m of the photic zone of a modern ferruginous basin^34^^,^^35^, enough to deposit the most voluminous BIF^36^. The ferric oxyhydroxides formed by these bacteria would have also provided protection from UV radiation when loosely attached to cell surfaces^37^.

Silica was another important component of Archean seawater, potentially reaching 2.2 mM^38^. Dissolved silica has a high affinity for ferric iron, and readily adsorbs onto the hydrous surfaces of iron oxides across a range of natural conditions^39,40^. Studies by Pierson et al.^41^ and Phoenix et al.^44^, using modern hot springs and experimental analogs, respectively, suggested that Fe(III)–silica gels and crusts overlying microbial mats could provide effective protection from UV-C radiation in Archean seawater. Here, building on this work, we explore the role of Fe(III)–silica precipitates in shaping the water column UV-C radiation profile and the resulting influence on cyanobacterial communities.

Through experimental analysis, we tested whether Fe(III)–Si precipitates forming in the Archean photic zone could have provided early planktonic cyanobacteria with an effective shield against UV-C radiation. It is impossible to sample the Precambrian biosphere directly, therefore, we rely on modern organisms as model systems. We explore these questions by growing the well-studied and fully sequenced marine cyanobacterium *Synechococcus* sp. PCC 7002 (hereafter *Synechococcus*) in Fe(III)- and Si-rich growth media simulating Archean seawater conditions^24,28,38^. We determined the effects on survival by exposing *Synechococcus* cultures to upper-end UV-C radiation fluxes (3 mW/cm^2^ at 254 nm)^3^, in experiments that were also used to understand UV radiation absorption by ancient seawater more generally. As per our experimental objective, UV-C radiation was chosen because, of the three classes of UV wavelength, it is the most harmful to life, and unlike today, it was an issue for inhabitants of Archean surface environments. DNA most readily absorbs at 260 nm and causes often lethal genetic damage (i.e., DNA strand breakage, mutagenic photoproducts such as cyclobutane pyrimidine dimers that inhibit transcription/replication of the chromosome)^11^. Low-pressure mercury lamps, such as the ones used in this study, are widely available and have a spectral peak at 254 nm—very close to DNA max absorbance, making this wavelength a reasonable choice to explore this problem. Furthermore, previous publications on protection from UV-C radiation by mat-forming cyanobacteria similarly used 254 nm, thus allowing comparison of attenuation effects in different modes of growth (i.e., planktonic vs. benthic)^42^. UV radiation fluxes were set to an order of magnitude higher than those predicted for the Archean (3 mW/cm^2^ compared with 0.4 mW/cm^2^)^3^ to provide a conservative estimate for the degree of efficiency with which these precipitates could protect cyanobacteria. Iron(III) (0.04–0.55 mM)^24,28^ and Si (0.6–2.0 mM)^38^ supplements were based on estimated Archean seawater concentrations, and we assumed a best case scenario of near-complete Fe(II) oxidation^34^.

Building from published geochemical constraints on Archean marine conditions and in-lab microbial growth and survival experiments, we were able to evaluate the extent of shielding from UV-C radiation that ferric iron precipitation in siliceous Archean seawater was likely to have provided for early planktonic cyanobacteria. We report that Fe(III)–Si precipitates suspended in the water column were able to absorb up to 70% of incident UV-C radiation while still transmitting the photosynthetically active radiation required for cyanobacterial growth. UV-C irradiated *Synechococcus* grown in iron–silica-rich growth media showed noticeably higher survival rates than irradiated cultures grown in un-supplemented media. However, we also observe that high mortality rates would have continued to limit cyanobacterial expansion, and suggest that these effects may have been felt throughout much of the Archean marine photic zone. These results imply that although Fe(III)–Si-rich precipitates would have played an important role in mitigating UV stress, this problem would not have been completely alleviated until atmospheric oxygen levels were sufficient to generate a protective ozone layer.

## Results

### UV-C irradiation experiments

**Fig. 1:**
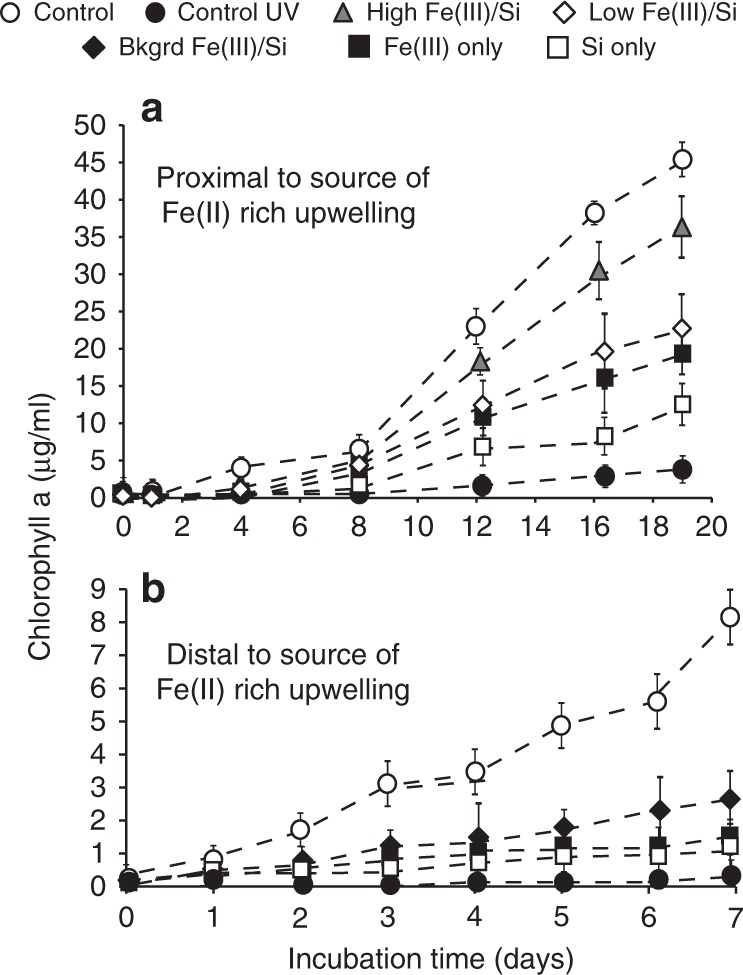
Growth rates of UV-C irradiated *Synechococcus* cultures. Cultures were irradiated at an exposure of 500 J/m^2^ in Fe(III)- and Si-supplemented media relative to irradiated (dark circle: control UV) and un-irradiated (white circle: control) cultures in un-supplemented growth media. **a** Fe(III) concentrations representing environments proximal to a source of iron-rich upwelling. White diamond: low Fe(III)/Si, 0.15/0.6 mM; gray triangle: high Fe(III)/Si, 0.55/2.0 mM; white square: Si only, 2.0 mM; black square: Fe(III) only, 0.35 mM. **b** Fe(III) concentrations (0.04 mM) representing environments located distally from a source of iron-rich upwelling (i.e., background concentrations for Archean seawater^25^). Black triangle: background Fe(III)/Si 0.04/0.6 mM. Growth rates calculated on cultures grown in triplicate in two biological replicates, error bars represent standard deviation at 2*σ* uncertainty

In this study, we test the ability of Fe(III) and Si to protect planktonic cyanobacteria from transitory exposure to high-intensity UV-C radiation. In the mixed layer of the modern photic zone, planktonic cells are at high risk of being transported to the ocean surface^17^, and we assume that this physical mechanism would also have existed in the Archean ocean. *Synechococcus* cultures exposed to UV-C radiation in Fe(III)- and Si-supplemented growth medium recover more rapidly from UV-C radiation stress than irradiated cultures in un-supplemented growth medium. In addition, growth rates increase steadily as initial Fe(III) and Si concentrations approach upper limits for Archean seawater (0.55 and 2.0 mM, respectively). Iron(III) or Si alone protect cells less efficiently from UV-C radiation than Fe(III) and Si combined. This is a crucial observation that implies that supplementation with both Fe(III) and Si is required for, and synergistically function as, a protective shield (Fig. 1a). This protective effect, though comparatively smaller, is also observed at Fe(III) concentrations of 40 μM. Lowermost Fe(II) concentrations for Archean seawater are predicted to range from 40 to 120 μM^28^, and assuming near-complete Fe(II) oxidation^34^, this may be a reasonable estimate for areas located distally from zones of iron-rich upwelling (Fig. 1b).

### Cell viability

**Fig. 2:**
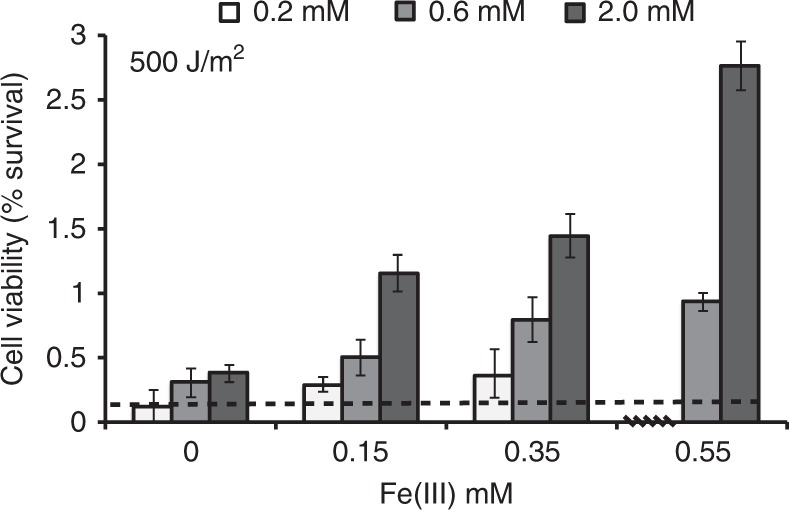
Viability of UV-C irradiated *Synechococcus* cultures. Cultures grown at varying Fe(III) (0 mM, 0.15 mM, 0.35 mM, and 0.55 mM) and Si concentrations (white bar: 0.2 mM Si; light gray bar: 0.6 mM Si; dark gray bar: 2.0 mM Si) and irradiated with UV-C (254 nm) at 500 J/m^2^ were assessed by colony forming units (CFU) counts. Cultures grown at 0.55 mM Fe(III) and 0.2 mM Si died (horizontal line). Viability was calculated for cultures grown in triplicate in three biological replicates. Error bars represent standard deviation at 2*σ* uncertainty. Dashed line represents % survival in un-supplemented A+ growth medium

The results reveal a positive correlation between initial Fe(III)–Si concentrations and cyanobacterial survival rates. However, cell viability assays show that UV-C irradiation at 500 J/m^2^ still dramatically reduce the population, with 97% mortality rate at the highest Fe(III)–Si concentrations. Survival increases from 0.13% viable cells in un-supplemented media to 2.76% at the highest Fe(III)–Si concentrations. In other words, the presence of Fe(III) and Si in the growth media facilitates the survival of only ~3% of the initial population following UV-C irradiation at 500 J/m^2^. This level is sufficient to seed a normal growth phase after 6 days (Fig. 2). Therefore, these results suggest that while Fe(III) and Si may have prevented high fluxes of UV radiation from sterilizing the early photic zone, UV radiation fluxes may still have had severe ecological impacts.

### Light transmission by Fe(III)–Si precipitates

**Fig. 3:**
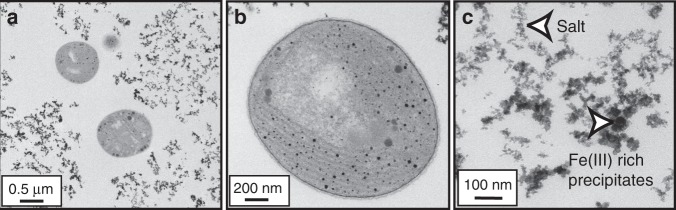
TEM imaging of *Synechococcus* cells and precipitates. **a** Transmission electron microscopy (TEM) image of nanoparticles surrounding (but not associated with) the cells in the presence of 0.35 mM Fe(III) and 2.0 mM Si. **b** TEM image showing the lack of biomineralization on *Synechococcus*. **c** Dark Fe(III)-rich precipitates and lighter salt nanoparticulates surrounding cells grown under conditions as shown in **a**

**Fig. 4:**
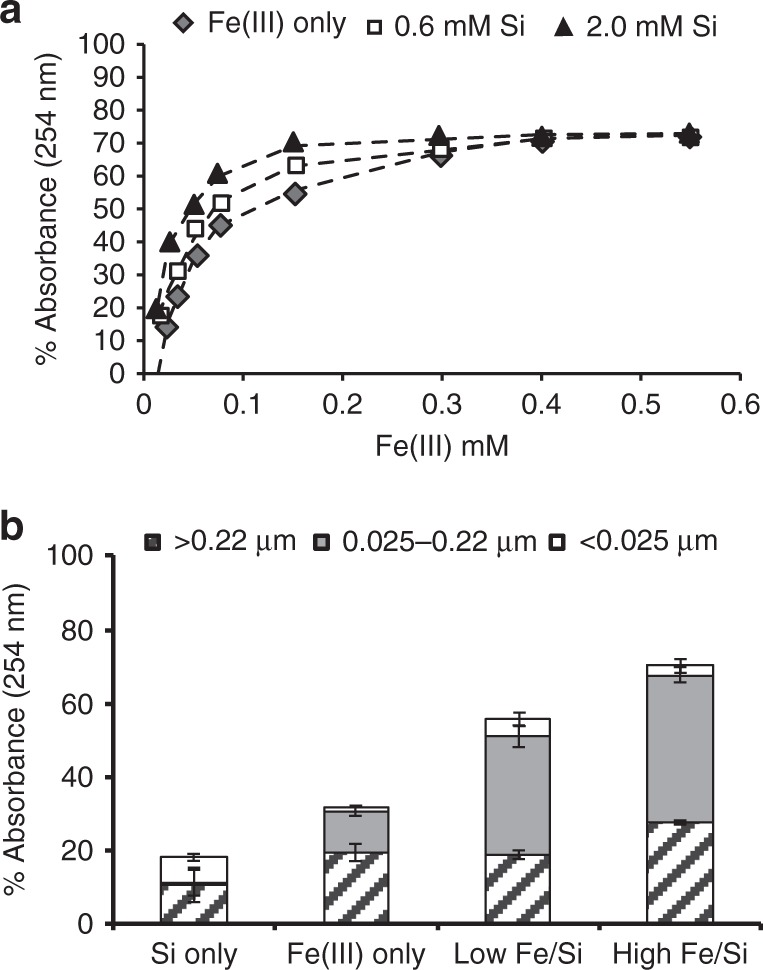
UV-C attenuation in cell free, Fe(III)–Si-supplemented growth media. **a** Attenuation (% absorbance at 254 nm wavelengths) of UV-C radiation in unfiltered, cell-free growth medium supplemented with varying amounts of Si and Fe(III) (diamond: Fe(III) only; square: 0.6 mM Si; triangle: 2.0 mM Si). **b** UV-C attenuation in solutions that have been sequentially filtered to separate dissolved (<0.025 μm), nanoparticulate (0.025–0.22 μm), and colloidal (>0.22 μm) Fe(III)–Si fractions. Attenuation effects of the A+ medium have been subtracted. Error bars (if not shown than smaller than symbols) represent standard deviation at 2*σ* uncertainty

Unlike previous studies in which mineral coatings provide protection, our samples display fine-grained colloidal precipitates surrounding, but not directly attached to the cells, with sizes in the tens-of-nanometer range (Fig. 3). The additive nature of the UV-C radiation absorbance curves and their comparable slopes in cell-free, Fe(III)–Si and Fe(III)-only media solutions indicate that Fe(III) is the primary factor that controls the attenuation of UV-C radiation (Fig. 4a). Furthermore, attenuation assays on the same supplemented, cell-free growth medium concur with the growth rate analyses indicating that the Fe(III)–Si supplementation differentially and progressively attenuates UV-C radiation by 18% in Si-only media, 32% in Fe(III)-only media, and by 56% and 70% in the lowest and highest Fe(III)–Si end-member ratios, respectively (Fig. 4b). These filtration analyses indicate that nanoparticles (0.025–0.22 µm) and colloids (>0.22 µm) in Fe(III)–Si containing growth media absorb up to 40% and 32% of incoming UV-C radiation, respectively, and thus performed a significant role in the enhancement of survival of cyanobacteria in the presence of UV-C radiation.

**Fig. 5:**
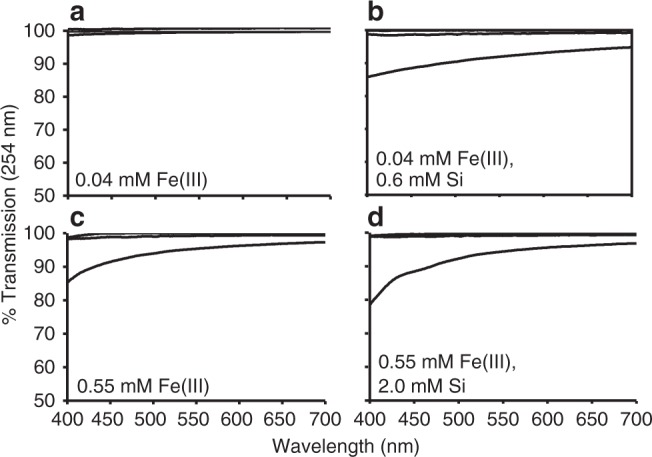
PAR transmission in cell-free, Fe(III)–Si-supplemented growth media. Transmission of photosynthetically active radiation (PAR: 400–700 nm; in %) through 0.04–0.55 mM Fe(III) (**a**–**d**) and 0.6–2.0 mM Si (**b**, **d**) supplemented growth media. Upper curve—dissolved. Middle curve—nanoparticulate/dissolved. Bottom curve—colloidal/nanoparticulate/dissolved. Similar transmission of UV-C radiation in dissolved and nanoparticulate/dissolved fractions as show by overlap of the upper and middle curves

The photosynthetic production of O_2_ by cyanobacteria depends on chlorophyll, phycocyanin, and carotenoid pigments that selectively absorb PAR^43^. Modern, clear, natural waters attenuate wavelengths outside the PAR range more readily through absorption^44^ and scattering by both dissolved and particulate matter^42^. Although Fe(III) and Fe(III)–Si precipitates larger than 0.22 µm absorb some incoming PAR at these concentrations, >80% of PAR is still transmitted through the supplemented media at high end-member ratios (Fe(III)/Si: 0.55/2.0 mM), while 99% of PAR is transmitted at low end-member ratios (Fe(III)/Si: 0.04/0.6 mM) (Fig. 5).

## Discussion

Our results demonstrate that although Fe(III)–Si precipitates would have moderated the impact of UV-C radiation stress on the survival of early cyanobacteria while not impeding cell growth, these UV-C radiation fluxes would have still limited cyanobacterial distribution within Archean marine photic environments. Similar to the presented results, biomineralized Fe(III)–Si crusts or gels encapsulating microbial mats are known to attenuate UV-C radiation while transmitting PAR in modern, benthic microbial communities^41,42^. As shown here and by others, Fe(III) on its own is known to be an efficient absorber of UV radiation especially at the shorter wavelengths^41^. The dominant form of the Fe(III) oxyhydroxide precipitates in our media is ferrihydrite as opposed to lepidocrocite, as ferrihydrite forms preferentially in solutions containing silica to iron ratios of 0.39 or higher (i.e., molar silica to iron ratios of experimental conditions here range from 1.9 to 9.3)^45^. The absence of solubility constants for polymeric Fe(III) and Fe(III)–Si complexes at circumneutral pH, as well as the amorphous nature of the precipitates, precludes accurate equilibrium speciation modeling of natural Fe(III)–Si-rich aqueous systems and X-ray fractionation for detailed particle characterization. Nonetheless, the formation of Fe(III)–Si precipitates has been characterized spectroscopically at lower pH^46^, and observations from the rock record strongly support the idea of a linked iron–silica cycle in the Archean^47–49^. Iron(III)–Si nanoparticles and colloids in Archean seawater would have formed as long as upwelling ferruginous waters and an oxidative mechanism persisted. Archean ferruginous waters likely occurred in both open ocean and coastal environments, as evidenced by thick successions of Neoarchean BIF interpreted to have been deposited in both deep and shallow marine settings^50,51^.

While we demonstrate that Fe(III) is the active UV radiation-absorbing ingredient in this system^41^, the major role of silica is to increase the stability of the colloidal and nanoparticulate ferrihydrite through the inhibition of Ostwald ripening^52^, thus delaying the formation of more stable Fe(III) (oxyhydr)oxides which would otherwise precipitate out of the water column. Silica will block dissolution sites or hinder the nucleation to more stable Fe(III) (oxyhydr)oxides at a pH > 3 by disrupting the corner-linkages between the iron-octahedra of the polymer, and by substituting itself into the second coordination-shell around the Fe(III) cation^53^. The effect of silica on colloidal stability has also been demonstrated experimentally in natural settings^54^. Longer colloidal settling times in a fluid occur as a function of increasing molar silica to iron ratio in solution^55^. Thus, in the absence of turbulence, published experimental work has established settling times of between 18 and 36 h in a saline solution for colloidal ferrihydrite formed under experimental molar silica to iron ratios comparable to the ones presented here^56^. These settling times, with flocculation being the dominant influence, would presumably increase in the presence of turbulence found in the mixed layer of the photic zone^57^. Multiple lines of evidence exist for pervasive co-precipitation of ferric iron and silica throughout the Archean water column^47–49^. Recent studies have suggested that the range of δ^56^Fe signatures found in Mesoarchean to Neoarchean aged BIF is best explained through isotopic fractionation during the co-precipitation of Fe(III) oxides and silica as well as through isotopic fractionation during the post-depositional, dissimilatory iron reduction of these precipitates^48,49^. In the modern Archean analog Lake Matano (Sulawesi, Indonesia), stable Fe(III) concentrations appear to be close to 100 nM—3 orders of magnitude lower than the aqueous Fe(II) concentration within the lake—while dissolved Si is only slightly lower than predicted for the Archean^28^. Therefore, further development of mechanistic models of Fe(III) removal from the water column at varying Fe(II) oxidation rates and Si concentrations are clearly required.

**Fig. 6:**
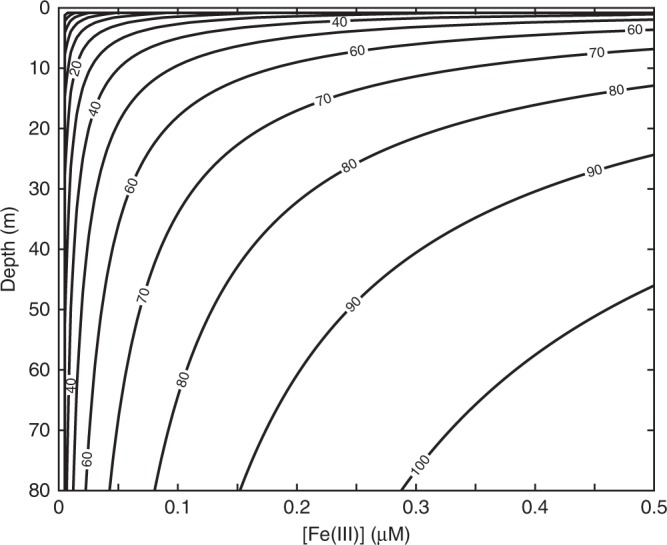
Reaction transport model of predicted UV-C radiation absorbance with water column depth and Fe(III) concentration. Model parameters are based on experimental UV-C radiation (254 nm) attenuation data (% UV-C absorbed) in cell-free A+ media solutions, and assume iron redox dynamics similar to those in modern Archean analog site Lake Matano (Sulawesi, Indonesia)^28^

If we scale up our experimental results to explore the penetration depth of a given UV radiation flux based on the effects of Fe(III)–Si absorbance, we can consider the potential for UV-C radiation effects over water column relevant depths. Based on the Beer–Lambert law, there should be a linear relationship between absorbance and concentration in an ideal solution. However, our experimental results indicate that other factors, including particle aggregation and suspended particles in solution, cause a roughly lognormal relationship. By approximating this data with a log function, we consider the predicted absorbance based on the experimental data for a given path length (depth) and concentration of Fe(III) (Fig. 6). This modeling framework indicates that at Fe(III) concentrations similar to that of the Archean analog Lake Matano (100 nM)^29^, absorbance of UV-C radiation is ~80% at 60 m depth. At the UV-C flux used in this study, this level of radiation would be detrimental to the survival of cyanobacteria (Figs. 2 and 4). It is important to note that Archean UV-C fluxes are predicted to have been lower than our experimental levels so as to not overestimate the degree of efficiency with which the precipitates could protect cyanobacteria. Nevertheless, the flux of UV-A and UV-B radiation reaching the Earth’s surface in the Archean are also predicted to have been several times higher than today^3^. Therefore, this exercise provides a basic, conservative, scaled-up result suggesting that although Fe(II) oxidation in siliceous seawater would have mitigated UV radiation stress, it is still likely to have been a factor shaping ecosystems and potentially even the rates of primary productivity. This is consistent with the longstanding idea^2^ that pervasive UV-C radiation within the Archean photic zone would have been a significant obstacle for early cyanobacteria.

An effective protection mechanism against UV radiation would have been necessary not only for the survival, but more importantly, the propagation and expansion of early communities of cyanobacteria. The attenuation of UV-C radiation in natural water occurs as a function of dissolved and particulate matter suspended in the water column, as well as by the absorptive properties of the water itself. Today, elevated levels of largely plant-derived CDOM is an important component in the attenuation of UV radiation, and occurs primarily in coastal marine waters^18^. In the Archean, however, terrestrial organic input into the oceans was likely substantially less than the modern^57^.

Stress caused by UV-C radiation is also known to reduce metabolic O_2_ production through downregulation of genes encoding proteins involved in light harvesting and photosynthesis, photobleaching of photosynthetic pigments (e.g., phycocyanin, phycoerythrin), disintegration of phycobilisomes, and decreasing cellular chlorophyll and carotenoid contents^11^. While the base of the mixed layer may have presented cyanobacteria with a deep refuge from UV radiation^2^, it enhances the potential for at least seasonal light limitation on primary production over significant expanses of the oceans—a condition currently observed in the Southern Ocean^58^. Further, microorganisms living within the mixed layer are often carried to the surface where, even in modern environments, short periods of exposure to UV radiation can be inhibitory^16^. Deep chlorophyll maxima are typically below the bottom of the mixed layer, with maximum depths of ~100–150 m in parts of the western north Pacific and western Atlantic oceans where PAR irradiance is on the order of only 3–5% of that at the ocean’s surface^2^. Finally, lower overall solar luminosities in the Archean^59^ would have also increased the potential for light limitation^60^.

While oxygenic photosynthesis may have evolved sometime during the Mesoarchean^22–24^, our results suggest that environmental factors such as UV radiation stress (particularly from short wavelengths) may have contributed to limited primary production by early cyanobacteria. Despite the various mitigation strategies that have evolved in modern cyanobacteria^7–11^, UV-A and UV-B radiation fluxes in the upper photic zone of the modern oceans still present a considerable challenge (UV-C is largely attenuated by the ozone layer)^16^. Therefore, even if early cyanobacteria had modern UV radiation defense and repair mechanisms at their disposal, the higher fluxes of UV radiation reaching the Earth’s surface (especially UV-C) would have limited their productivity until a more efficient means of protection developed. An ozone layer would have been the most effective and permanent means of protection from UV-C radiation, but would not have fully developed until O_2_ levels reached ~1% PAL^1,3^, which likely did not occur until the mid-Proterozoic at the earliest as current estimates for mid-Proterozoic levels range from 0.1 to 10% PAL^61,62^.

Building on this framework, a major conclusion from this work is that despite the high Fe(III) and Si concentrations enabling their survival, UV-C irradiation still could have induced high rates of mortality for planktonic communities of cyanobacteria in the upper reaches of the water column. Extrapolating beyond Earth, these limiting effects have implications for the potential of light-limited exoplanetary biospheres^5,6^. While Fe(III)–Si particles and colloids contributed to the mitigation of limited ozone concentrations, the presence of these chemical species would not have alleviated the UV radiation problem completely, as has been suggested for benthic communities^41,42^. Thus, future Archean global biogeochemical models should work to directly integrate the effects of UV irradiation on the delineation and organismal composition of the biosphere.

## Methods

### Microorganism

The marine planktonic cyanobacterium *Synechococcus* sp. PCC 7002 (previously known as *Agmenellum quadruplicatum*)^63^ is a unicellular, euryhaline, marine cyanobacterium. It was chosen as the model organism due to its adaptation to higher light conditions and dissolved iron levels compared with other strains of *Synechococcus* and *Prochlorococcus*^64,65^. The laboratory wild-type strain was originally obtained from Professor Donald Bryant at Pennsylvania State University.

### Culturing

Axenic cultures were grown in A+ medium^66^: 0.31 M NaCl; 8 mM KCl; 12 mM NaNO_3_; 20 mM MgSO_4_•7H_2_O; 0.37 mM KH_2_PO_4_; 2.4 mM CaCl_2_; 0.08 mM Na_2_EDTA•2H_2_O; 0.014 mM FeCl_3_•6H_2_O; 8.25 mM Tris HCl (pH 8.2); P-1 metals (0.55 mM H_3_BO_4_; 22 µM MnCl_2_•4H_2_O; 2.3 µM ZnCl_2_; 0.22 µM MoO_3_ (85%); 0.012 µM CuSO_4_•5H_2_O; 0.051 µM CoCl_2_•6H_2_O); and 59 nM vitamin B12 prepared in 18.2 MΩ cm MilliQ water. See Supplementary Note 1 for a discussion on the effects of EDTA on Fe(III) and Si reactivity in A+ growth medium (Supplementary Fig. 1). Axenic stock populations were maintained on 1.5% (w/v) A+ agar in Petri dishes at 30 °C under constant illumination (~50 µmol photons/m^2^/s). Cultures for inoculating experiments were prepared from agar-based cultures by suspension of cyanobacteria into 50 mL of media in 250 mL borosilicate glass Erlenmeyer flasks with gas-permeable foam stoppers (Canlab Dispo Plugs). In order to obtain sufficient biomass for Fe(III) and Si growth experiments from a single culture, 300 mL cultures were grown in 1 L borosilicate glass Erlenmeyer flasks with gas-permeable foam stoppers and bubbled with sterile air to enhance CO_2_ supply. The 14% v/v inoculum used to establish these 300 mL cultures was derived from a single 50 mL liquid culture. The 300-mL culture was then used to inoculate 50 mL experimental cultures in A+ medium supplemented with concentrations of Si as Na_2_SiO_3_ (to final Si concentrations of 0.2 mM, 0.6 mM, and 2.0 mM) and Fe(III) as FeCl_3_•6H_2_O in the A+ media (to final Fe(III) concentrations of 0.04 mM, 0.15 mM, 0.35 mM, and 0.55 mM). Control experiments (un-irradiated cultures grown in A+ medium with no added iron and silica) were also included, as well as cultures grown in Fe(III)-only supplemented A+ medium and Si-only supplemented A+ medium to ascertain the effects of these compounds individually on growth rate. Cultures were grown in triplicate, and experiments repeated in two independent biological replicates. Stock cultures for inoculating experiments were harvested when optical density measurements at a wavelength of 750 nm (OD_750_) indicated that they had reached mid-exponential growth phase (~4 × 10^6^ cells/mL).

The oxic conditions used for these experiments do not preclude the main premise of this study, to examine whether Fe(III)–Si precipitates suspended in the water column could protect planktonic cyanobacteria from UV-C radiation. As evidenced in the BIF record, Fe(III) oxides were ubiquitous in the Archean water column. Once cyanobacteria had evolved, the microenvironment proximal to cells of cyanobacteria would have contained free O_2_^22–24^. And although the mechanisms of Archean Fe(II) oxidation prior to the evolution of oxygenic photosynthesis are still debated, it has been argued that photoferrotrophs metabolically oxidizing Fe(II) in the ancient photic zone were the most plausible producers of Fe(III), although Fe(II) photo-oxidation and reaction with hydrogen peroxide, albeit less productive processes, were other potential mechanisms^30,31^.

### UV irradiation

To ascertain the effects of silica and ferric iron during the UV irradiation of live cells, cultures were irradiated in a Stratalinker UV Crosslinker 1800 containing 5 × 254 nm (8 W) bulbs collectively emitting radiation at 3 W/cm^2^ (Supplementary Fig. 2). Experimental cultures were harvested at mid-exponential phase (OD_750_ indicating ~4 × 10^6^ cells/mL), where 10 mL were transferred into sterile plastic Petri dishes (no lid) using a sterile glass pipette so that the entire surface was covered by a thin layer of liquid (Supplementary Fig. 3). Cultures were irradiated at 500 J/m^2^ (i.e., 3 mW/cm^2^ for 16.7 s) followed by a 6-h dark incubation. The selected dose of UV-C radiation was based on maximal depression of cell viability in un-supplemented cultures (Supplementary Fig. 4). Samples were then grown at 30 °C under full spectrum fluorescent light (Philips F48T12/CW/HO 60 W) where wavelengths < 520 nm were filtered out (Roscolux Supergel filter plastic #15) to prevent photoreactivation of nucleic acids damaged by UV radiation. These measures were taken to ensure that the data represented growth and viability as a direct result of UV radiation damage. Incubation periods varied from 7 to 19 days to ascertain patterns of immediate recovery as well as long-term viability and growth.

### Determination of cell viability post-UV irradiation

Total chlorophyll *a* concentration was used as proxy for growth rate^67^, and cultures were sampled in duplicate every day or every second day for chlorophyll *a* and OD_750_ measurements. As a measure of cell viability in irradiated samples, the ratio of live to dead cells was used and determined by counting the number of colony forming units (CFU). Appropriately diluted aliquots (100 µL) of irradiated, dark incubated samples were plated on A+ agar and subsequently incubated under optimal light conditions (with filtered wavelengths < 520 nm to prevent photoreactivation, as described above) for 10 days. When grown at a temperature of 30 °C in A+ medium, a cell suspension with OD_750_ = 1 typically contained 6.3 ± 0.4 × 10^8^ cells per mL (*n* = 3) as determined by microscopic counts, and a count of 2.1 ± 0.6 × 10^8^ cfu per mL (*n* = 3). Each treatment was done in triplicate, and repeated in three biological replicates.

### Light transmission and particle size characterization

The attenuation of UV-C radiation (254 nm) and photosynthetically active radiation (PAR: 400–700 nm) by Fe(III)- and Si-supplemented growth media was determined in triplicate on a Pharmacia Ultrospec 3000 spectrophotometer using quartz (for UV-C) and polystyrene (for PAR) cuvettes with 1 cm path lengths. Attenuation by various Fe(III)–Si size fractions were also determined by filtering media solutions twice using cellulose acetate Millipore filters, firstly to 0.22 µm, whereupon the attenuation of UV-C radiation at 254 nm and PAR transmission was measured. The resulting filtrate was subsequently filtered to 0.025 µm and measurement attenuation of UV-C radiation at 254 nm and PAR transmission repeated. Results for the various size fractions were determined by normalizing results to those of unfiltered, un-supplemented solutions in order to take into account the effects of total dissolved solids in the saline medium.

### Transmission electron microscopy

For transmission electron microscopy (TEM) imaging of the cells, *Synechococcus* cultures grown in Fe(III)- and Si-supplemented A+ media were fixed in a solution of 2.5% gluteraldehyde + 2% Paraformaldehyde, dehydrated through an ascending ethanol series (50–100%), stained with 1% osmium tetroxide, embedded in Spurr low viscosity resin, thin sectioned (80–100 nm) using a Reichert-Jung Ultracut E microtome, and sections stained with 2% uranyl acetate. For images of the Fe(III)–Si precipitates, a drop of cell-free media was spread onto a cellulose film supported by a copper grid. TEM images were obtained using a Philips/FEI Morgagni 268 TEM operating at 80 kV equipped with a Gatan Orius Digital Camera using Gatan Digital Micrograph version 1.81.78 imaging software.

### UV-C penetration estimates

Our estimate for the penetration depth of UV-C radiation was based directly on experimental results, and therefore reflects an estimate for monochromatic penetration of UV radiation at 254 nm. A log function was fit to the measured absorbance of UV-C radiation 0.6 mM Si resulting from changing concentrations of Fe(III) (Fig. 4). Because the absorbance of UV-C radiation was measured over a 1 cm path length, we considered the moles of Fe(III) present over a given path length, and compared that to percentage absorbance predicted by our experimentally-derived log function for the same number of moles within 1 cm^3^ volume.

### Data availability

The data sets generated during and/or analyzed during the current study are available from the corresponding authors on reasonable request.

## Electronic supplementary material

Supplementary Information
