# Supplementary material for: UV radiation limited the expansion of cyanobacteria in early marine photic environments
Source: Nat Commun. 2018 Aug 6;9:3088. doi: 10.1038/s41467-018-05520-x (PMC6079077; doi:10.1038/s41467-018-05520-x)
Supplement: Supplementary file 1 — Supplementary Information [file 41467_2018_5520_MOESM1_ESM.pdf]

SUPPLEMENTARY INFORMATION: UV radiation limited the expansion of cyanobacteria in early marine photic environments

Mloszewska *et al.*

Supplementary Figure 1. Modeled chemical equilibrium concentrations for A+ growth medium. Mineral saturation indices (IAP/Ksp) as a function of silicic acid (H_4SiO_4) have been added to the model. Regular A+ medium is saturated with respect to ferrihydrite [$(Fe^{+3})_2O_3$•$0.5H_2O$], while the remainder of the Fe(III) is found as soluble organic Fe(III)-EDTA and soluble inorganic Fe(III) complexes. According to the model, the addition of Fe(III) to the media simply increases the amount of ferrihydrite precipitate. The equilibrium solubility model was calculated using Visual MINTEQ version 3.05, where the default MINTEQ thermodynamic database was updated to include stability constants for aqueous the Fe(III)-Si complex $FeOSi(OH)_3^{+2}$ as compiled by Pokrovski et al. (2003)[46]. Modeling parameters included (i) standard room temperature (25ºC), (ii) supersaturated minerals were permitted to precipitate, (iii) salinity was determined by the ionic strength of the solution, and (iv) the activity coefficients were calculated using the Davis Equation. The calculations were performed by fixing equilibrium with respect to excess calcite ($CaCO_3$) and atmospheric pCO_2.

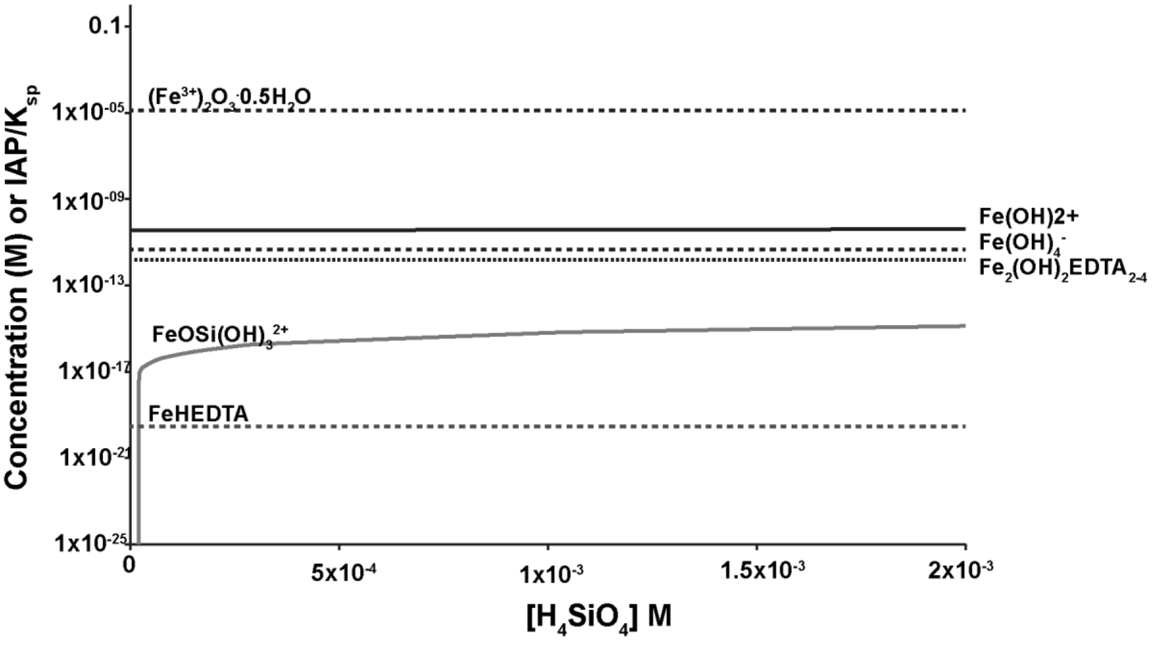

Supplementary Figure 2. Dimensions of the UV irradiation setup. Experimental cultures were irradiated in a Stratalinker UV Crosslinker 1800 containing 5x 254nm bulbs collectively emitting radiation at 3 mW/cm^2.

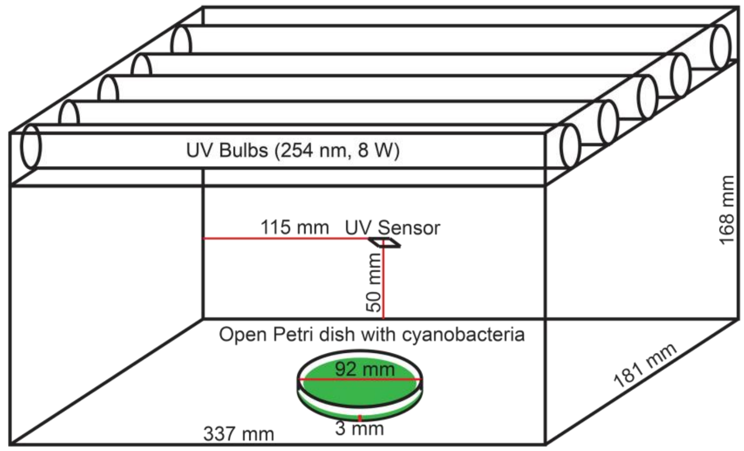

Experimental Set-up inside
Stratalinker UV Crosslinker (Model 1800)

Supplementary Figure 3. **Irradiation setup in the Stratalinker UV Crosslinker 1800.** Experimental cultures were transferred into sterile plastic Petri dishes (no lid) using a sterile glass pipette so that the entire surface was covered by a thin layer of liquid.

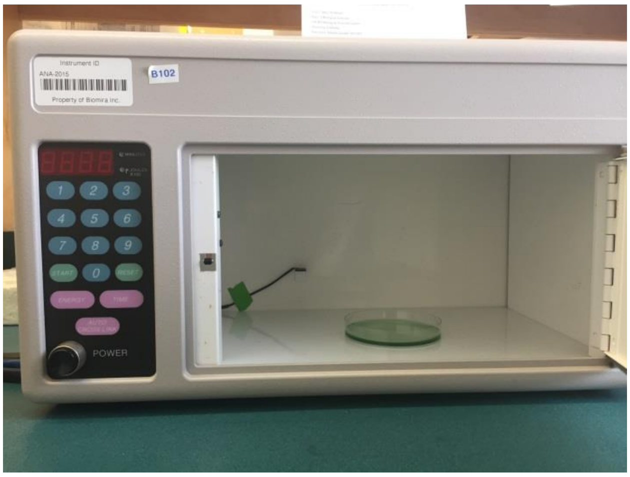

Supplementary Figure 4. Growth trials for irradiated *Synechococcus* sp. PCC 7002 in un-supplemented media. *Synechococcus* cultures grown in A+ media were irradiated with UV-C (254 nm) doses ranging from 100 J/m^2 to 1000 J/m^2. OD_{750} represents cellular growth rates over the number of days post incubation. The UV-C dose chosen for the UV experiments in this study (500 J/m^2) were based on the maximal depression of cellular growth rates on un-supplemented cultures in A+ growth medium while still allowing sufficient cell growth to enable reasonable cell mass for analyses over reasonable incubation periods.

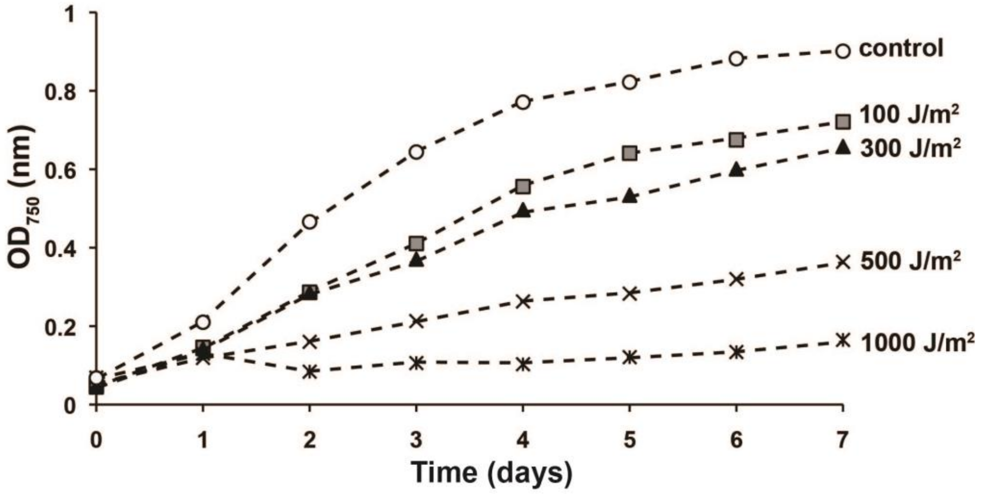

Supplementary Note 1

A+ growth medium[66] contains 0.08 mM disodium ethylenediaminetetraacetic acid (Na_2EDTA), an organic ligand used to sequester metals such as Fe(III). Its role is to facilitate the uptake of essential metals that would not be bioavailable to bacteria otherwise. We modelled the effects of Na_2EDTA on Fe(III) at normal concentrations in A+ medium (14.4 µM)[66]. At this concentration, the media is saturated with respect to the mineral ferrihydrite - $(Fe^{+3})_2O_3$•$0.5H_2O$, where the addition of more Fe(III) only increases the amount of this mineral that should precipitate. Aqueous, monomeric Fe(III)-Si complexes are a minor player at circumneutral pH (maximal formation occurs at pH 3, Pokrovski et al., 2003)[46]. At circumneutral pH, dimer, trimer and higher polymeric Fe(III)-Si species should form[46]. However, the absence of equilibrium constants available for these aqueous polymeric Fe(III)-Si species at circumneutral pH precludes any accurate geochemical modeling of their concentrations. In terms of the breakdown of Na_2EDTA complexation in A+ medium, the majority (~80%) of the Na_2EDTA is bound to the Ca (2.4 mM) and Mg (20 mM) in the A+ media, whereas around 6% of it is bound to the Fe(III).
